# Developmental delay in Rett syndrome: data from the natural history study

**DOI:** 10.1186/1866-1955-6-20

**Published:** 2014-07-22

**Authors:** Jeffrey L Neul, Jane B Lane, Hye-Seung Lee, Suzanne Geerts, Judy O Barrish, Fran Annese, Lauren McNair Baggett, Katherine Barnes, Steven A Skinner, Kathleen J Motil, Daniel G Glaze, Walter E Kaufmann, Alan K Percy

**Affiliations:** 1 Department of Pediatrics, Baylor College of Medicine, Houston, TX 77030, USA; 2 Duncan Neurological Research Institute, Texas Children's Hospital, Houston, TX 77030, USA; 3 Civitan International Research Center, University of Alabama at Birmingham, 1720 2nd Avenue South, Birmingham, AL 35294-0021, USA; 4 Pediatrics Epidemiology Center, University of South Florida, Tampa, FL 33620, USA; 5 Greenwood Genetic Center, Greenwood, SC 29646, USA; 6 Boston Children’s Hospital, Harvard Medical School, Boston, MA 02115, USA

## Abstract

**Background:**

Early development appears normal in Rett syndrome (OMIM #312750) and may be more apparent than real. A major purpose of the Rett Syndrome (RTT) Natural History Study (NHS) was to examine achievement of developmental skills or abilities in classic and atypical RTT and assess phenotype-genotype relations in classic RTT.

**Methods:**

Developmental skills in four realms, gross and fine motor, and receptive and expressive communication from initial enrollment and longitudinal assessments for up to 7 years, were assessed from 542 females meeting criteria for classic RTT and 96 females with atypical RTT divided into two groups: 50 with better and 46 with poorer functional scores. Data were analyzed for age at acquisition and loss of developmental features and for phenotype-genotype effects. Acquired, lost, and retained skills were compared between classic RTT and atypical RTT with better or poorer functional scores using Fisher's Exact test. To examine if the mean total score from the Motor Behavioral Assessment during follow-up differed for acquiring a skill, we used a generalized estimating equation assuming compound symmetry correlation structure within a subject. A general linear model was used to examine whether the mean age of acquisition or loss of a developmental skill differed by mutation type. *P* values <0.05 were considered significant and were two-sided without adjustment for multiple testing. Statistical analyses utilized SAS 9.3 (SAS Institute, Cary, NC, USA).

**Results:**

Early developmental skills or abilities were often acquired albeit later than normal. More complex motor and communication acquisitions were delayed or absent. Clinical severity was less in those achieving the respective skill. Individuals with R133C, R294X, and R306C point mutations and 3′ truncations tended to have better developmental outcomes.

**Conclusions:**

Early developmental skills were acquired by many, but clear differences from normal emerged, particularly in skills expected after age 6 months. When comparing clinical severity, greater acquisition of specific skills was associated with specific mutations, confirming the impression that these mutations confer milder developmental abnormalities. These data may serve for planning and interpretation of early intervention studies in RTT.

**Trial registration:**

This NHS study, clinicaltrials.gov (NCT00296764), represents the largest group of RTT participants assessed repeatedly by direct examination.

## Background

Rett syndrome (RTT), OMIM #312750, a neurodevelopmental disorder predominantly affecting females, has been characterized by ‘apparently’ normal initial development followed by frank regression of fine motor and communication skills typically between 6 and 18 months of age [1-3]. Despite absence of prospective evidence of delayed early development, retrospective review has suggested that abnormalities are evident within the first 6 months [4-7]. Infants have often been described as being hypotonic and occasionally being excessively irritable or having postural stiffness often belying the underlying hypotonia. Assessments of development have been hampered by relatively small numbers of participants, possibly allowing phenotypic variability to skew assessments, involved data derived from questionnaires without direct assessment of the participants by clinicians experienced in the diagnosis of RTT, or focused on specific skills or time periods rather than acquisition of developmental skills longitudinally [4-10]. Videotaped assessments have provided important retrospective observations with regard to specific early developmental skills. These prior studies indicate that the early period of development in RTT could be regarded as abnormal [6,7,10] and evidence of abnormal deceleration in head growth occurring as early as age 1.5 months based on recent data from the NICHD-sponsored Rare Disease Natural History Study (NHS) provides neuroanatomical support [11].

Information obtained over the past 7 years through the NHS has yielded extensive longitudinal data on a large cohort of individuals with classic and atypical RTT, providing definitive evidence for developmental patterns regarding the achievement of specific milestones that deviate from normal. Here, we capture and compare the acquisition of specific skills or abilities and whether these fall within the limits for achieving accepted milestones for the respective skills or abilities. Further, we extend the relationship between these developmental trajectories and specific *MECP2* mutations and compare these trajectories in participants with classic and atypical RTT.

## Methods

Data from initial enrollment in the NHS were assessed from 542 females who met criteria for classic RTT and 96 females who met criteria for atypical RTT and were enrolled into the study at an initial age under 10 years old. Although our overall cohort of individuals with RTT exceeded 900, we chose to restrict the analysis to subjects seen initially before 10 years of age in order to increase accuracy of parental recall for the acquisition of developmental skills or abilities (in the following sections, we will use the term ‘skills’ for both). Age at enrollment for this group ranged from 7 months to 10 years of age; median age was 4 for classic RTT, 4.5 for higher function atypical RTT, 3.5 for lower function atypical RTT. As individuals with atypical RTT have a bimodal distribution, those less severely affected and those more severely affected, this group was divided into 50 having better functional scores (clinical severity score ≤20) and 46 having poorer functional scores (clinical severity score >20). Criteria for enrollment were based on clinical assessment by experienced clinicians, requiring each participant (1) to fulfill consensus criteria for RTT [12,13] and (2) to have genetic testing for the responsible gene, *MECP2* (*Methyl-CpG-binding protein 2*), although identification of a *MECP2* mutation was not essential for inclusion. Nevertheless, 530 (97.8%) of 542 classic RTT participants and 83 (86.5%) of 96 atypical RTT participants had a *MECP2* mutation.

Developmental achievement of specific skills was obtained in four categories through a detailed, direct interview with the parents or responsible caregivers based on specific recall aided by baby books and pictures, association with key events or time points such as birthdays, holidays, or other celebrations, and the review of prior medical evaluations by primary care physicians and any subspecialists: gross motor, fine motor, receptive communication, and expressive communication. In regard to developmental categories, parents were asked to provide information in three phases: the specific age at which the skill was acquired; whether and at what age it was lost; and whether and at what age it was regained. These participants were evaluated every 6 months if less than 6 years of age and annually thereafter. For the majority of this cohort, 337 (62%) of participants with classic RTT and 66 (69%) with atypical RTT were below age 6 at the time of enrollment. Developmental data were reviewed for each participant at semi-annual if less than age 6 or annual visits.

The present report is restricted to acquisition, loss, and overall retention of skill. Retained skills were determined as follows: Acquired skill − Loss of skill + Regained skill/Group total = % Retained skill. For classic RTT, specific ages could be assigned to each skill in >95% and for atypical RTT, in greater than 97%. The remaining data could not be recalled. These were updated, particularly for children <3 years of age, at periodic exams conducted semi-annually for the first 5 years of age and annually thereafter. Clinical severity scores (CSS) [14] and motor behavioral assessments (MBA) [15] were acquired at each visit and results compared with each developmental achievement. The CSS assessed the ordinal scores in 13 domains (age at regression, age at stereotypy onset, degree of deceleration of head growth, growth (BMI) status, sitting, walking, hand function, scoliosis, vocalization/verbalization, eye contact, periodic breathing, hand/foot skin temperature, and seizures). Total score range was 0 to 58 with higher scores representing greater clinical severity. The MBA examined 37 ordinal scores in three domains: behavioral-social (16 items), orofacial/respiratory (7 items), and motor/physical (14 items). Total score range was 0 to 148, with higher scores representing greater severity.

### Statistical analysis

The study group consisted of 542 classic RTT and 96 atypical RTT. The atypical RTT group was further subdivided as noted above. Whether or not the proportion of acquiring, losing, or retaining a developmental skill was different among groups is summarized in Tables 1 and 2. The proportion of lost skills was Number lost/Number who acquired; the proportion of overall retention of skills was the Number retained/The group total. Skills for acquired, lost, and retained were compared between classic, atypical better, and atypical poorer using Fisher's Exact test. To examine if the mean total score of the MBA during follow-up was different for acquiring a skill, we used a generalized estimating equation assuming compound symmetry correlation structure for all total scores of MBA measured within a subject, after adjusting for age at enrollment, in each diagnosis group (Tables 3 and 4). Data related to a regained skill were too sparse for a separate analysis.

**Table 1:** Acquired developmental milestones: gross and fine motor

**Skill**	**Classic (*N*** **= 542, 5 without answers)**			**Atypical better (*N*** **= 50)**			**Atypical poorer (*N*** **= 46, 1 without answers)**		
	**Acquired**	**Lost**	**Retained**	**Acquired**	**Lost**	**Retained**	**Acquired**	**Lost**	**Retained**
Gross motor									
Rolling	95 (511)	23 (118)	77 (414)	96 (48)	6.3 (3)*	96 (48) **◊**	87 (39) ●	28 (11)	62 (28)*
Sit with support	97 (520)	12 (62)	88 (473)	100 (50)	2.0 (1)●	100 (50)	93 (42)	19 (8)	84 (38)
Sit alone	80 (427)	16 (70)	69 (369)	94 (47)*	0 (0)	94 (47) **◊**	31 (14) **◊**	21 (3)	24 (11) **◊**
Crawl	69 (370)	38 (139)	45 (244)	88 (43)*	7.0 (3) **◊**	82 (41) **◊**	22 (10) **◊**	40 (4)	13 (6) **◊**
Pull to stand	62 (331)	34 (112)	43 (231)	92 (46) **◊**	4.3 (2) **◊**	88 (44) **◊**	16 (7) **◊**	43 (3)	8.9 (4) **◊**
Walk with help	79 (422)	13 (54)	71 (383)	94 (47) *	4.3 (2)	92 (46) **◊**	31 (14) **◊**	14 (2)	27 (12) **◊**
Walk alone	53 (284)	14 (41)	47 (253)	78 (39) **◊**	13 (5)	70 (35) **◊**	6.7 (3) **◊**	67 (2)	2.2 (1) **◊**
Climb Steps	20 (106)	26 (27)	15 (81)	62 (31) **◊**	23 (7)	52 (26) **◊**	0 (0)	0 (0)	0 (0)
Ride tricycle	4 (22)	32 (7)	3.2 (17)	16 (8)*	0 (0)	16 (8) *	2.2 (1)	0 (0)	2.2 (1)
Fine motor									
Hold bottle	85 (455)	49 (223)	46 (248)	98 (49)*	12 (6) **◊**	92 (46) **◊**	71 (32) ●	34 (11)	53 (24)
Reach	97 (523)	49 (255)	58 (314)	100 (50)	10 (5) **◊**	94 (47) **◊**	91 (41) ●	27(11)*	76 (34)*
Transfer	78 (418)	61 (257)	69 (370)	96 (48) **◊**	17 (8) **◊**	86 (43) **◊**	71 (32)	38(12)*	49 (22)*
Pincer grasp	74 (396)	72 (285)	24 (128)	90 (45)*	36(16) **◊**	66 (33) **◊**	42 (19) **◊**	32 (6) **◊**	29 (13)
Finger feed	91 (489)	56 (276)	45 (242)	96 (48)	10 (5) **◊**	90 (45) **◊**	64 (29) ●	28 (8)*	47 (21)

% (*N* = number): *p* values for Classic vs. Atypical better and Classic vs. Atypical poorer are indicated for acquired, lost, and retained skills ●<0.05; *<0.01; ◊<0.001.

**Table 2:** Acquired developmental milestones: expressive and receptive language

**Skill**	**Classic (*N*** **= 542, 5 without answers)**			**Atypical better (*N*** **= 50)**			**Atypical poorer (*N*** **= 46, 1 without answers)**		
	**Acquired**	**Lost**	**Retained**	**Acquired**	**Lost**	**Retained**	**Acquired**	**Lost**	**Retained**
Receptive communication									
Fix and follow	94 (503)	28 (143)	86 (464)	94 (47)	8.5 (4)*	94 (47) ●	89 (40)	7.6 (3)*	85 (39)
Quiet to voice	85 (457)	26 (118)	77 (416)	84 (42)	7.1 (3)*	84 (42)	71 (32)*	0 (0)	71 (32)
Inhibit to no	66 (357)	25 (88)	56 (303)	76 (38)	7.9 (3) ●	72 (36)*	47 (21)*	10 (2)	42 (19) ●
Follow command with gesture	55 (295)	36 (107)	42 (225)	74 (37)*	16 (6)*	68 (34) **◊**	29 (13) **◊**	7.7 (1)*	27 (12) ●
Follow command with no gesture	45 (241)	32 (76)	36 (195)	70 (35) **◊**	20 (7)	62 (31) **◊**	24 (11)*	9.1 (1)	22 (10) ●
Expressive communication									
Social smile	99 (535)	16 (85)	96 (518)	100 (50)	10 (5)	100 (50)	98 (44)	14 (6)	96 (43)
Coo	93 (499)	36 (178)	67 (360)	98 (49)	14 (7)*	86 (43) **◊**	78 (35)*	31 (11)	56 (25)
Babble	95 (511)	51 (259)	64 (346)	98 (49)	18 (9) **◊**	90 (45) **◊**	80 (36) **◊**	33 (12)	62 (28)
Single words	77 (412)	86 (354)	21 (113)	94 (47)*	55 (26) **◊**	68 (34) **◊**	36 (16) **◊**	13 (2) **◊**	36 (16) ●
Phrases	18 (98)	76 (74)	5.0 (28)	54 (27) **◊**	41 (11) **◊**	42 (21) **◊**	6.7 (3)	0 (0)	6.7 (3)
Gestures	53 (286)	86 (246)	10 (56)	70 (35)*	46 (16) **◊**	44 (22) **◊**	31 (14)*	50 (7)*	18 (8)
Points for wants	23 (121)	65 (79)	9.5 (51)	58 (29) **◊**	28 (8) **◊**	44 (22) **◊**	4.4 (2)*	100 (2)	0 (0) **◊**

% (*N* = number): *p* values for Classic vs. Atypical better and Classic vs. Atypical poorer are indicated for acquired, lost, and retained skills ●<0.05; *<0.01; ◊<0.001.

**Table 3:** Motor-behavioral assessment and acquired developmental milestones: gross and fine motor

**Developmental skill**	**Parameter estimate ± Standard error, from the generalized estimating equation after adjusting for age at enrollment (Skill not gained vs. gained)**		
	**Classic**	**Atypical better**	**Atypical poorer**
Rolling	7.9 ± 2.6 (*p* = 0.003)	6.9 ± 4.6 (*p* = 0.132)	5.9 ± 5.4 (*p* = 0.274)
Sit with support	14.8 ± 2.6 (*p* < 0.0001)	N.A.	2.2 ± 2.9 (*p* = 0.453)
Sit alone	8.7 ± 1.0 (*p* < 0.0001)	10.5 ± 2.3 (*p* < 0.0001)	3.5 ± 2.9 (*p* = 0.237)
Crawl	8.4 ± 0.9 (*p* < 0.0001)	8.7 ± 3.1 (*p* = 0.005)	−0.8 ± 3.2 (*p* = 0.796)
Pull to stand	9.3 ± 0.9 (*p* < 0.0001)	12.3 ± 2.0 (*p* < 0.0001)	2.6 ± 2.8 (*p* = 0.358)
Walk with help	11.0 ± 1.0 (*p* < 0.0001)	12.3 ± 2.8 (*p* < 0.0001)	1.7 ± 3.0 (*p* = 0.577)
Walk alone	10.9 ± 0.8 (*p* < 0.0001)	9.0 ± 2.4 (*p* = 0.0001)	2.1 ± 4.6 (*p* = 0.647)
Climb steps	11.2 ± 1.2 (*p* < 0.0001)	6.4 ± 2.8 (*p* = 0.023)	N.A
Ride tricycle	10.6 ± 3.0 (*p* = 0.0004)	7.3 ± 3.0 (*p* = 0.015)	12.3 ± 2.6 (*p* < 0.0001)
Hold bottle	6.4 ± 1.1 (*p* < 0.0001)	−2.0 ± 2.6 (*p* = 0.432)	3.4 ± 2.9 (*p* = 0.245)
Reach	6.5 ± 2.7 (*p* = 0.015)	N.A.	2.6 ± 3.6 (*p* = 0.483)
Transfer	4.2 ± 1.1 (*p* < 0.0001)	9.8 ± 2.7 (*p* = 0.0003)	7.4 ± 2.8 (*p* = 0.008)
Pincer grasp	6.0 ± 1.0 (*p* < 0.0001)	10.7 ± 2.5 (*p* < 0.0001)	2.8 ± 2.7 (*p* = 0.304)
Finger feed	8.3 ± 1.4 (*p* < 0.0001)	16.1 ± 2.2 (*p* < 0.0001)	0.8 ± 2.3 (*p* = 0.744)

**Table 4:** Motor-behavioral assessment and acquired developmental milestones: expressive and receptive communication

**Developmental skill**	**Parameter estimate ± Standard error, from the generalized estimating equation after adjusting for age at enrollment (Skill not gained vs. gained)**		
	**Classic**	**Atypical better**	**Atypical poorer**
Fix and follow	−2.3 ± 2.2 (*p* = 0.287)	8.4 ± 3.0 (*p* = 0.005)	2.7 ± 6.4 (*p* = 0.679)
Quiet to voice	1.6 ± 1.5 (*p* = 0.269)	4.3 ± 3.2 (*p* = 0.184)	7.5 ± 3.3 (*p* = 0.025)
Inhibit to no	5.2 ± 1.0 (*p* < 0.0001)	1.6 ± 2.9 (*p* = 0.586)	5.1 ± 2.5 (*p* = 0.038)
Follow command with gesture	4.1 ± 0.9 (*p* < 0.0001)	6.8 ± 2.6 (*p* = 0.010)	0.1 ± 3.2 (*p* = 0.972)
Follow command with no gesture	3.5 ± 1.0 (*p* = 0.0002)	4.2 ± 2.6 (*p* = 0.102)	−2.4 ± 3.0 (*p* = 0.430)
Social smile	10.0 ± 2.9 (*p* = 0.0007)	N.A.	1.7 ± 1.6 (*p* = 0.299)
Coo	2.6 ± 1.8 (*p* = 0.152)	13.2 ± 2.4 (*p* < 0.0001)	−2.3 ± 2.8 (*p* = 0.423)
Babble	3.0 ± 2.2 (*p* = 0.165)	−2.3 ± 2.8 (*p* = 0.410)	6.4 ± 3.9 (*p* = 0.097)
Single words	6.3 ± 1.1 (*p* < 0.0001)	7.0 ± 1.8 (*p* = 0.0001)	3.0 ± 2.3 (*p* = 0.194)
Phrases	7.3 ± 1.2 (*p* < 0.0001)	2.9 ± 2.5 (*p* = 0.248)	11.8 ± 1.7 (*p* < 0.0001)
Gestures	2.8 ± 0.9 (*p* = 0.003)	6.5 ± 2.4 (*p* = 0.007)	3.1 ± 2.3 (*p* = 0.191)
Points for wants	7.2 ± 1.1 (*p* < 0.0001)	8.1 ± 2.3 (*p* = 0.0004)	4.4 ± 6.4 (*p* = 0.490)

Among the 542 females with classic RTT, we examined whether the mean age of acquisition or loss of a developmental skill differed by mutation type. Mutations were analyzed as severe (R106W, T158M, R168X, R255X, R270X, and large deletions) and mild (R133C, R294X, R306C, and 3′ truncations) [14,16]. A general linear model was used (Additional file 1).

**Figure 1:**
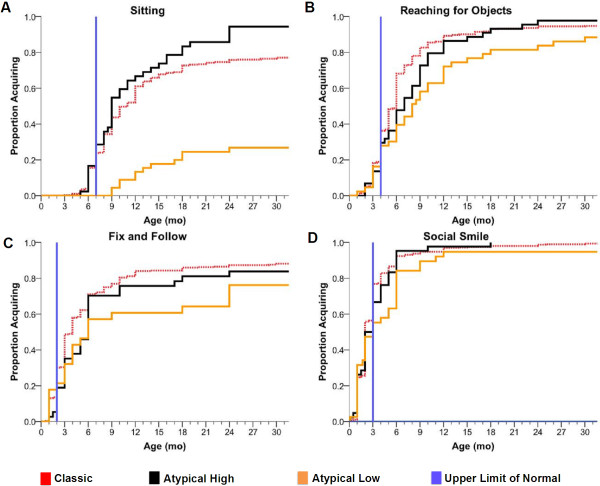
**Inverse Kaplan-Meier graphs were created for acquired developmental milestones.** Expected upper limits of normal are represented by the vertical blue line [17]. These plots represent the early four skills, **(A)** sitting alone, **(B)** reaching, **(C)** fixing and following, and **(D)** social smile. The remaining acquired skills and the Kaplan-Meyer graphs for lost skills are displayed as additional files. In each case, more than 95% (517/542) of classic RTT and more than 97% (93/96) of atypical RTT participants reported a specific age of acquisition for each skill.

Graphs were created depicting the temporal pattern for twelve primary elements of acquired and lost developmental skills. For each, an upper limit of normal for the accepted milestone was noted based on standards reported by Feigelman [17]. Figure 1a,b,c,d is included in the manuscript reflecting plots for sitting, reach for objects, fixing and following, and social smile. The remaining eight graphs for acquired features and the 12 plots for lost features are included as Additional file 2: Figure S1 and Additional file 3: Figure S2.

*P* values less than 0.05 were considered significant. All reported *P* values are two-sided without adjustment for multiple testing. Statistical analyses were performed using SAS 9.3 (SAS Institute, Cary, NC, USA).

### Human studies approval

Each site obtained and maintained IRB approval for the performance of this study. Parental approval for study conduct and publication of results was obtained before entry into the study. The study has been registered with ClinicalTrials.gov: NCT00299312 since 3 March 2006.

## Results

Data for acquisition of developmental skills were derived from 542 participants with classic RTT and 96 with atypical RTT, all less than 10 years of age at the initial assessment. The atypical RTT group displayed a bimodal distribution of clinical severity resulting in two groups representing better (50) or poorer (46) CSS. This atypical RTT grouping resulted in two distinct patterns of involvement and specific *MECP2* mutations (Table 5). The CSS value for classic RTT had a mean of 21.8 (range 7 to 43; SD 6.81); for atypical better, the CSS value was 12.0 (range 3 to 20; SD 4.60), and for atypical poorer, the CSS value was 27.6 (range 21 to 39; SD 4.15). Mutations in the better functioning group were concentrated among three point mutations (R133C, R294X, and R306C) and 3' truncations, representing 58% (29/50). Conversely, 15.2% (7/46) of individuals in the poorer functioning group had one of these mutations. The poorer functioning group had more than twice as many lacking a *MECP2* mutation. In both instances, this distribution differed from the classic RTT group (*p* = 0.0004) where the eight common point mutations comprise 59% and 3' truncations, large deletions, and other insertions/deletions represent 25% of the total.

**Table 5:** ***MECP2 *** **Mutations in classic and atypical RTT groups**

**Mutation**	**Classic Rett syndrome**	**Atypical Rett syndrome**	
	**% ( *N *)**	**Better function % ( *N *)**	**Poorer function % ( *N *)**
None	2.2 (12)	8.0 (4)	19.5 (9)
R133C (397C > T)	4.6 (25)	16.0 (8)	0 (0)
R306C (916C > T)	7.6 (41)	10.0 (5)	2.2 (1)
R294X (880C > T)	4.8 (26)	6.0 (3)	0 (0)
R270X (808C > T)	5.7 (31)	2.0 (1)	8.7 (4)
R255X (763C > T)	11.4 (62)	2.0 (1)	17.4 (8)
R168X (502C > T)	12.2 (66)	0 (0)	8.7 (4)
T158M (473C > T)	9.7 (53)	2.0 (1)	4.4 (2)
R106W (316C > T)	3.0 (16)	0 (0)	6.5 (3)
3′ Truncations	8.7 (47)	26.0 (13)	13.0 (6)
Large deletions	8.8 (48)	4.0 (2)	8.7 (4)
Other point mutations	11.6 (63)	20.0 (10)	6.5 (3)
Insertions/deletions	7.8 (42)	2.0 (1)	4.4 (2)
Exon 1	0.6 (3)	2.0 (1)	0 (0)
Splice site	1.3 (7)	0 (0)	0 (0)
Total	100 (542)	100 (50)	100 (46)

Nearly all participants in all diagnostic groups acquired early gross motor skills such as rolling and sitting with support (Table 1); however, other gross motor skills were less likely to be acquired. In classic RTT, the percentage of participants acquiring a particular skill decreased as the skill became more advanced. A minority of participants was able to achieve the most advanced skills such as climbing steps or riding a tricycle. The atypical groups had distinct patterns of gross motor skill acquisition compared with classic RTT. The better functioning atypical group did not show the decline in percentage of individuals gaining more advanced skills, with nearly 90% acquiring skills up to walking with help. In contrast, the poorer functioning atypical group showed an even sharper decline in percentage of participants gaining gross motor skills, with a minority gaining the ability to sit alone or other more advanced skills.

Among fine motor skills in the classic group, reaching for an object and finger feeding were most likely to be acquired whereas pincer grasp or transfer were noted in 74% to 78%. As with gross motor capabilities, participants with classic RTT were significantly better than the poorer functioning atypical group whereas the higher functioning atypical group did better than those with classic RTT.

Loss of acquired motor skills occurred in all groups. In classic RTT, fine motor skills were more likely to be lost than gross motor skills (Table 1). The better functioning atypical group showed a similar pattern of greater fine motor than gross motor loss; however, the percent losing either skill was far less than in classic RTT. This ultimately led to a greater percentage of those with better functioning atypical RTT demonstrating retained motor skills. The poorer functioning atypical group had similar loss of both gross and fine motor skills. In comparison to classic RTT, a similar percentage of this poor functioning group retained fine motor skills but fewer retained gross motor skills.

Early expressive communication skills (social smile, coo, babble) were attained by nearly all classic and better functioning atypical participants, but in a smaller percentage of poorer functioning atypical individuals (Table 2). Although a large percentage of classic RTT participants gained single words, only a minority developed phrases. In contrast, nearly all better functioning atypical participants gained single-word skills and over 50% developed phrases. The atypical poorer functioning group uniformly acquired fewer expressive communication skills than the classic RTT group. Babble was lost in a significant fraction of classic RTT, and more advanced expressive communication skills (single words, phrases) were lost in nearly all. Conversely, loss of expressive language skills was not as dramatic in atypical better functioning participants, a large fraction retaining these skills. As noted for motor skills, participants with classic RTT attained expressive communication skills intermediate between individuals with better and poorer functioning atypical groups (Table 2). In general, receptive communication skills were retained far better than expressive communication skills for all groups and better for the better atypical group than for classic RTT.

An important question is how the acquisition of development skills relates to overall functioning. To assess this, we determined the effect of not gaining a skill on the MBA change during follow-up using a generalized estimating equation adjusted for age at enrollment and diagnosis. Notably, not acquiring a motor skill at the time of enrollment resulted in worse overall functioning over time, as shown by an increase in the MBA score (Table 3). This effect was seen both at the baseline visit as well as subsequent visits. When the skill was not acquired, those with classic RTT tended also to have had worse MBA scores than individuals in the better functioning atypical group, but had generally better MBA scores than the poorer functioning group. When the MBA scores were adjusted for age at enrollment and diagnosis, whether or not a motor skill was acquired was highly significant for each motor skill at baseline and for subsequent visits for classic RTT and for most skills for atypical RTT (Table 3).

For receptive and expressive communication, the MBA scores revealed a quite similar pattern, namely individuals with classic RTT had worse MBA scores than the better functioning group but better MBA scores than the poorer functioning group. Acquisition of early communication skills such as social smile, cooing, fixing and following, and quieting to voice did not affect age adjusted MBA scores (Table 4). For more complex skills such as single words, gestures, or inhibiting to ‘No’ or following commands, MBA scores were significantly better in general for those acquiring the specific skill (Table 4).

In classic RTT, acquisition of developmental skills was achieved in many participants; however, the timing of skill acquisition was delayed compared with skill acquisition in typically developing children (Figure 1, Additional file 2: Figure S1, and Additional file 3: Figure S2). Only a minority of participants achieved most skills at the expected time. Graphs depicting the temporal pattern of skill acquisition and the respective milestone revealed definitive patterns of abnormality. Figure 1a,b,c,d demonstrates that sitting, reaching for a toy, and fixing and following were acquired by the expected age in 30% to 35% while social smile was acquired in about 75%. Other features were acquired between 30% to 60% of normal (Additional file 2: Figure S1). Graphs depicting the loss of acquired skills in classic RTT also revealed variable patterns. Gross motor and receptive communication abilities were lost by fewer participants than fine motor and expressive communication (Additional file 3: Figure S2). These data provide additional evidence for subnormal acquisition of developmental skills and greater loss of acquired skills in specific realms.

Previous studies in classic RTT have shown that individuals with R133C, R294X, R306C, and 3' truncations have less significant involvement than individuals with T158M, R168X, R255X, and R270X [14,16,18]. Examining these mutations individually did not demonstrate significant differences, perhaps because of too few participants. When mutations were compared by two groups, severe (R106W, T158M, R168X, R255X, R270X), and large deletions versus mild (R133C, R294X, R306C, and 3' truncations, the severe group showed a smaller proportion in acquired skills only for following commands with a gesture and a tendency for transferring objects (Additional file 1). However, a greater proportion lost skills compared to the mild group. Among the lost features, differences (Additional file 1) were noted in gross motor skills (come to sit and walking with support), fine motor skills (hold bottle, transfer, pincer grasp, and finger feeding, receptive language (fix and follow), and expressive language (babble, single words, phrases, gestures, and point for wants).

## Discussion

Early development in individuals with RTT has been regarded as ‘apparently’ normal although significant questions have been raised [4-10]. The present study involving 542 participants with classic RTT and 96 with atypical RTT demonstrated that early developmental skills are acquired in most but not all participants and clear differences emerged, particularly regarding whether specific skills were acquired within the expected norms. When comparing MBA, individuals who attained specific developmental skills tended to have lower (better) scores than those who did not. With regard to specific skills, gross motor skills were retained better than fine motor skills for the most part, and receptive communication better than expressive communication. When individuals with atypical RTT were separated into two groups in terms of neurologic function (i.e., high or better and low or poorer), and compared with classic RTT, a distinctive pattern appeared. Participants with classic RTT displayed an intermediate profile between the better and poorer functioning atypical groups with respect to acquisition and loss of skills.

Analysis of atypical participants led to the first formal identification of two subtypes of individuals (i.e., better and poorer functioning), beyond the well-defined entities such as preserved speech and early seizure variants. Comparison between better and poorer functioning atypical groups showed clear differences regarding distribution of mutation types. Those with better functioning atypical RTT such as those with preserved speech patterns tended to be clustered among three specific point mutations and 3' truncations whereas those with poorer functioning atypical RTT such as those with very early onset developmental impairment were more broadly arrayed among mutation groups and clustered among common point mutations with poorer overall CSS scores. This distribution represented a dramatic difference from either those with classic or the better functioning atypical RTT.

Phenotype-genotype correlation studies have shown that individuals with specific *MECP2* mutations tend to have overall better neurologic function and developmental skill profile than others. This study reinforced the concept that individuals with R133C, R294X, R306C, and 3' truncations acquire more gross motor skills and lose fewer skills, particularly in fine motor and expressive language. Yet, we have learned from other studies [19,20] that these individuals may have greater behavioral issues in terms of anxiety, aggressiveness, and inappropriate activities. However, as other phenotype-genotype studies have shown, specific mutations may not be the only determinant of severity within specific individuals due to the existence of other factors such as X chromosome inactivation, genetic background (the interplay of other genetic variations), and distribution of the abnormal gene in specific brain regions [14,16,18,21].

The pattern of acquisition and loss of skills supports the notion that in RTT, developmental progression predicts overall level of function. This is also reflected in the developmental profile of specific *MECP2* mutations. The abnormal acquisition of early skills, specifically those acquired before 6 months, is in line with the highly prevalent head deceleration that begins in early postnatal life [11]. Both features suggest a pathogenic process that affects developmental events taking place shortly after birth. Whether these events correspond to arrest or involution in synaptic development needs to be determined. The observation that two subtypes of atypical RTT exist based on skill profiles and overall severity is a significant result. Atypical RTT may not be a milder or more severe form of RTT reflecting a greater or lesser severity of MeCP2 deficit, but instead reflects a qualitatively different pathogenic process. We hope that this study stimulates more investigations regarding the phenotypic spectrum of atypical RTT.

The present study was based on data from a white population to a significant extent. While every effort was expended to assess a truly representative dataset, this shortcoming must be kept in mind. Another limitation is that the developmental data were obtained retrospectively. The majority of the participants was less than age 6 at the time of enrollment, but data were provided by informants up to 10 years after birth for the remainder. Review of primary care records and association with key time points were utilized to improve data retrieval Assessments of videos taken by parents during critical developmental periods may facilitate in demonstrating early motor and communication abnormalities in RTT [6-10,22]. This approach could be used in the future for validating developmental milestone data during parental interviews more systematically. The existing video reports related to early development are based on small numbers of participants that might not cover the complete spectrum of abilities. Therefore, a more comprehensive inclusion of videotaped assessments could be a valuable approach. However, as the diagnosis is often delayed by a number of years, prospective analysis of this type might be difficult to apply.

The data presented here do reflect both cross-sectional and longitudinal perspectives in RTT and should provide critical information for clinicians and therapists as well as for stratification in future clinical trials for this very challenging neurodevelopmental disorder.

## Conclusions

Early developmental skills in RTT are acquired by many, but clear differences emerge in skills expected after 6 months of age. The early developmental skills develop nearly uniformly but often the age at acquisition is delayed beyond the normal period to achieve the accepted milestone limits. Confirmation and extension of phenotype-genotype relations is provided noting that R133C, R294X, R306C, and 3' truncations are generally associated with milder delays. This large cohort of participants assessed directly by experienced clinicians for up to seven years provides a complete sequence of developmental skill or ability acquisition, loss, and retention. Furthermore, the recognition of individuals with atypical RTT as representing a mixture of two groups with quite different patterns of skill acquisition and retention suggests a qualitatively different pathogenic process may exist.

## Abbreviations

BMI: body mass index; CSS: clinical severity score; *MECP2*: *Methyl-CpG-binding protein 2*; MBA: motor behavioral assessment; NICHD: National Institute of Child Health and Human Development; NHS: Natural History Study; RTT: Rett syndrome.

## Competing interests

The authors declare that they have no competing interests.

## Authors’ contributions

All authors have been involved in drafting or revising the manuscript, have given final approval, and agree to be accountable for all aspects of the work involved. Each author's individual participation is outlined below. JLN and AKP did the conceptualization and design of the study and acquisition, analysis, and interpretation of the data. H-SL did the statistical analysis of the data. SAS, KJM, WEK, SG, JOB, LMB, and KB did the acquisition and interpretation of the data. DGG and JBL did the conceptualization and design of the study and acquisition and interpretation of the data.

## Authors’ information

The RTT Natural History Study team composed of authors with significant experience in RTT has worked together since 2006 to acquire and analyze the data. This work involves semi-annual or annual visits at which time all elements of the history and examination are updated providing both cross-sectional and longitudinal data. This study represents the largest single study in which clinical data are acquired in exactly this manner.

## Supplementary Material

Additional file 1 Supplemental table. Click here for file

Additional file 2 Inverse Kaplan-Meier graphs for nine acquired developmental skills (A. Sitting, B. Pull to stand, C. Walking, D. Transfer, E. Pincer grasp, F. Quiet to Voice, G. Inhibit to No, H. Babbling, I. Single words). Click here for file

Additional file 3 Kaplan-Meier graphs for twelve lost developmental skills A. Sitting, B. Pull to stand, C. Walking, D. Transfer, E. Pincer grasp, F. Reaching, G. Inhibit to ‘No’, H. Babbling, I. Single words, J. Social smile, K. Fix and follow, L. Quiet to voice. Click here for file
