# Supplementary material for: Developmental delay in Rett syndrome: data from the natural history study
Source: J Neurodev Disord. 2014 Jul 22;6(1):20. doi: 10.1186/1866-1955-6-20 (PMC4112822; doi:10.1186/1866-1955-6-20)
Supplement: Additional file 1 — Supplemental table. [file 1866-1955-6-20-S1.docx]

**Supplemental Table**

**Analysis of Acquisition and Loss of Developmental Skill vs. Mutation Group**

**Group I vs. II: Parameter estimate (Standard error), p-value**

|  | **Acquisition of Developmental Skill** | **Loss of Developmental Skill** |
| --- | --- | --- |
| **Milestone** |  |  |
| Roll front to back | 0.445(0.412), 0.281 | -8.631(4.701), 0.070 |
| Sit when placed | 0.035(0.565), 0.950 | -10.70(7.529), 0.162 |
| Come to Sit | 0.267(0.640), 0.677 | -20.52(6.497), 0.003 |
| Crawl | -0.355(0.719), 0.622 | -6.470(3.367), 0.058 |
| Pull to stand | 0.601(0.736), 0.414 | -6.235(3.278), 0.061 |
| Walk with support | 0.732(1.164), 0.530 | -14.03(6.575), 0.039 |
| Walk independently | 1.673(1.211), 0.169 | 5.750(8.891), 0.523 |
| Stairs up alone | 0.555(3.829), 0.885 | -10.00(7.261), 0.192 |
| Pedal tricycle | -12.67(9.888), 0.229 | N.A. |
| Hold bottle | -1.028(0.580), 0.077 | -10.40(2.238), <0.0001 |
| Reach for toy | -0.182(0.953), 0.848 | -1.903(1.917), 0.322 |
| Transfer | -1.486(0.765), 0.053 | -4.941(1.758), 0.006 |
| Pincer grasp | -1.237(0.917), 0.178 | -6.086(1.460), <0.0001 |
| Finger feed | -0.222(0.990), 0.823 | -3.838(1.872), 0.042 |
| Fix and follow | -0.488(1.595), 0.760 | -4.515(1.412), 0.002 |
| Quiet to voice | 1.013(1.347), 0.453 | -2.588(2.278), 0.259 |
| Inhibit to ‘No’ | -2.576(1.790), 0.152 | -2.690(2.724), 0.328 |
| Follow command with gesture | -4.946(2.449), 0.045 | -3.027(2.158), 0.165 |
| Follow command without gesture | -3.938(2.914), 0.179 | -1.728(3.399), 0.614 |
| Social smile | -0.569(0.563), 0.313 | -4.696(2.402), 0.056 |
| Coo | 0.381(0.348), 0.274 | -1.502(1.833), 0.415 |
| Babble | 0.283(0.542), 0.602 | -4.688(1.526), 0.003 |
| Single words | -0.629(0.649), 0.333 | -2.780(1.111), 0.013 |
| Phrases | -5.058(3.950), 0.206 | -7.313(2.553), 0.006 |
| Gestures | 0.300(1.481), 0.840 | -2.814(1.245), 0.025 |
| Points for wants | -1.054(3.838), 0.785 | -5.920(2.495), 0.022 |

Group I contains T158M, R168X, R255X, R106W, R270X, and large deletions (severe)

Group II contains R133C, R294X, R306C, and 3’ truncations (mild)
