# Supplementary material for: Developmental delay in Rett syndrome: data from the natural history study
Source: J Neurodev Disord. 2014 Jul 22;6(1):20. doi: 10.1186/1866-1955-6-20 (PMC4112822; doi:10.1186/1866-1955-6-20)
Supplement: Supplementary file 2 [file 1866-1955-6-20-S2.docx]

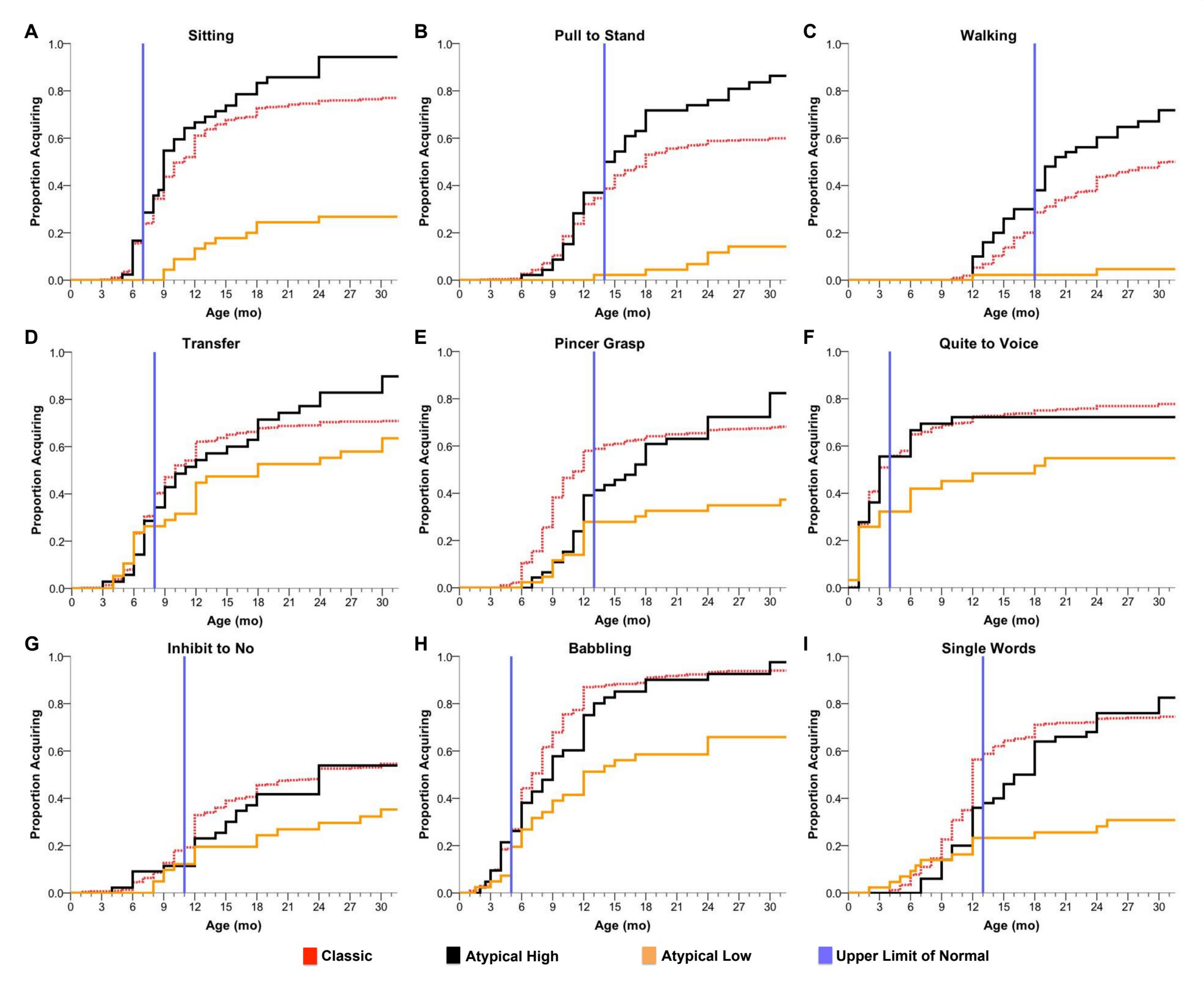
A
Sitting
B
Pull to Stand
C
Walking
D
Transfer
E
Pincer Grasp
F
Quite to Voice
G
Inhibit to No
H
Babbling
I
Single Words
Proportion Acquiring
Age (mo)
Classic
Atypical High
Atypical Low
Upper Limit of Normal
